# Computational Strategies for Predicting Excited‐State Energies in Eu^3+^ Down‐Shifting Spectral Converters for Photovoltaic Devices

**DOI:** 10.1002/cphc.202500543

**Published:** 2025-11-23

**Authors:** Laura Sánchez‐Muñoz, Daniel Aravena, Jordi Cirera, Pere Alemany

**Affiliations:** ^1^ Departament de Ciència de Materials i Química Física and Institut de Recerca de Química Teòrica i Computacional Universitat de Barcelona (IQTCUB) Diagonal 645 08028 Barcelona Spain; ^2^ Materials Chemistry Department Faculty of Chemistry and Biology Universidad de Santiago de Chile Avenida Libertador Bernardo O’Higgins 3363 Santiago 1058 Chile; ^3^ Departament de Química Inorgànica i Orgànica and Institut de Recerca de Química Teòrica i Computacional Universitat de Barcelona (IQTCUB) Diagonal 645 08028 Barcelona Spain

**Keywords:** antenna effect, density functional theory, emission, lanthanides, time‐dependent density functional theory

## Abstract

In this work, a computational protocol has been developed to predict the ligand‐based low‐lying excited‐state energies of Eu^3+^ coordination compounds with antenna ligands. A computational strategy, based on density functional theory (DFT) and time‐dependent density functional theory (TD‐DFT), has been developed using compounds with reliable structural and spectroscopic experimental data as a reference set. This approach aims to predict both the geometry and energy of the lowest‐excited triplet state, critical factors influencing the efficiency of the antenna effect and energy transfer to the Eu^3+^ ion. The model not only shows the ability to replicate available experimental data at a relatively low computational cost, but also accurately predicts triplet‐state energies for compounds that have not been included in the training set. This work is a first step toward the development of an affordable method for accurate predictions of the quantum yield of lanthanide‐based complexes to assess their potential application as down‐shifting spectral converters in solar cells.

## Graphical abstract

A computational strategy to compute the energy of the triplet excited state in europium‐based coordination complexes is presented. This method allows for in silico screening of new systems with can be used as photosensitizers in solar cell‐based technologies.

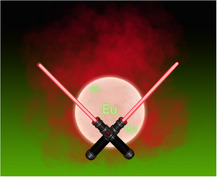

## 1 Introduction

Photovoltaic (PV) devices, directly converting sunlight to electricity, are a key factor in the transition to a green economy. One of the main problems in the massive use of PV devices is, however, their low power conversion efficiencies due to the mismatch between the incoming solar spectrum and the absorption profile of the cell.^[^ ^1^ ^]^ Despite being a highly prevalent issue in the research and development of new strategies to improve the efficiency of PV devices, the mismatch between the solar spectrum and the absorption range of the PV cell has not yet been satisfactorily solved.^[^ ^2^ ^]^ A recent approach to improve the efficiency of PV cells involves the use of spectral converters, materials that absorb radiation in a given wavelength range and reemit it as photons with wavelengths in the range where the PV cell's absorption is optimal.^[^ ^3^ ^]^

Lanthanide cations (Ln^3+^) are of great technological interest due to their long average radiative lifetimes, which makes them highly interesting to develop new materials for applications such as organic light‐emitting diodes, lasers, biomarkers, or sensors, among others.^[^ ^4^, ^5^, ^6^, ^7^ ^]^ Some Ln^3+^ ions have strong emissions in the UV–visible range (Ce^3+^, Sm^3+^, Eu^3+^, Tb^3+^, and Dy^3+^). In the case of Eu^3+^, this ion has an intense emission within the bandgap region of silicon (1.1 eV = 1127 nm), making it a promising choice as a down‐shifting spectral converter for the widespread crystalline silicon (c‐Si) solar cells. Currently, the most studied complexes for this purpose are those formed with Eu^3+^, followed by those with Tb^3+^.^[^ ^8^, ^9^, ^10^ ^]^ These compounds can be used in down‐shifting layers (Luminiscent down‐shifting layers), which can be applied directly to the surface of the PV cell to convert UV light into wavelengths that are more efficiently absorbed by the silicon‐based active layers, enhancing the photocurrent generation.^[^ ^11^ ^]^ Although lanthanide cations have the highest quantum yields for spectral conversion, they have low absorption coefficients in the UV–vis spectral region since f–f transitions are forbidden by Laporte's selection rule.^[^ ^12^ ^]^ However, their performance can be improved by including the Ln^3+^ cation in coordination compounds using antenna ligands, which can act as sensitizers.^[^ ^13^ ^]^

Over the years, several compounds have been synthesized and their photophysical data measured. Besides this, some computational work has been performed to unravel the details of the mechanism behind the antenna effect of their ligands in order to single out the factors leading to compounds with high quantum yields.^[^ ^14^, ^15^, ^16^ ^]^ This search has, however, been far from systematical, but developed rather for individual compounds and using different computational approaches, which hinders the development of a reliable and precise method for estimating the quantum yield at an affordable computational cost.

**Figure 1:**
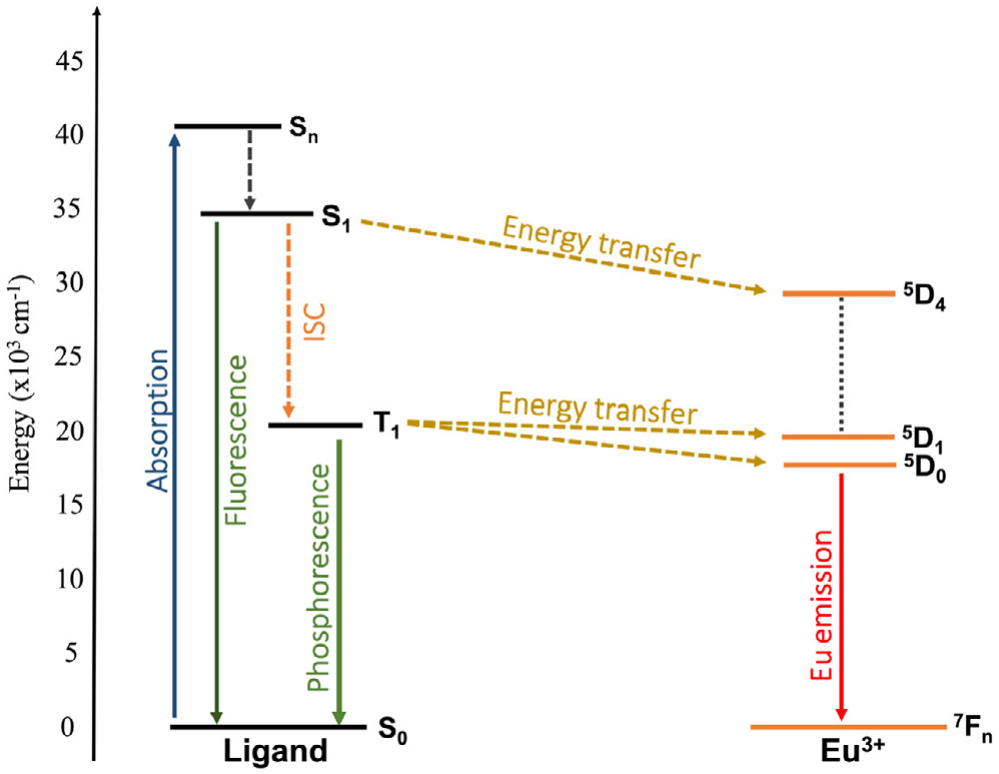
Jablonski diagram for a Eu^3+^ complex with an antenna ligand. Radiative transitions are represented with solid arrows, and nonradiative ones with dashed arrows.

Having a robust method to compute the photophysical parameters involved in the antenna effect is a fundamental tool for designing new efficient compounds. The present research is focused on Eu^3+^ complexes with organic ligands that absorb UV–visible radiation (*λ*_ex_ = 300–600 nm) present in solar radiation, converting it to useful low‐energy photons (*λ*_em_ = 1127 nm for the case of Eu^3+^), in what is known as a down‐shifting process, but can be easily extended to other lanthanide cations. In these molecular compounds, the absorption and excitation are mainly localized within a single ligand, and as a result, the efficiency of energy transfer depends critically on the specific position of the ligand's lowest‐excited triplet state, which must match one of the Ln^3+^ excited states for an efficient energy transfer from the ligand to the central Ln^3+^ ion, as shown in **Figure** **1**.

Following Latva's empirical rule,^[^ ^17^ ^]^ the ligand's lowest triplet energy level *T*_1_ needs to be ≈2.500 cm^−1^ above the ^5^D_0_ excited state of Eu^3+^ to ensure an energy transfer from the ligand to the europium cation. Since the emissive states of Eu^III^ lay within the visible range (580.48 nm/17.227 cm^−1^), the optimal energy transfer window between the ligand and the metal ion ranges between 19.000 and 23.000 cm^−1^. For this reason, any screening protocol aiming to develop new Eu‐based complexes to be used as spectral converters must be able to estimate the energy of the ligand's lowest‐excited triplet state and check its alignment with the Eu^3+^ ^5^D_0_ level. This alignment is, however, only a necessary condition for a good energy transfer between the ligand and the central Eu^3+^ cation. One also needs to consider further features of the complex, such as the number and coordination geometry of the metal center, the type of donor atoms, and the nature of the metal–ligand interactions, among others. These factors ensure an efficient energy transfer from the ligand to the lanthanide cation, allowing for the prediction of the quantum yield for the corresponding process.

The main objective of the present work is to develop a reliable computational strategy to a) determine the geometry of Eu^3+^ complexes with antenna ligands, both in their singlet ground state and in their lowest excited ligand‐based triplet state and b) predict the values of the triplet‐excited‐state energy of the ligands in the complexes at a reasonable computational cost to include them as a first screening step in a computational tool for designing new Eu^3+^‐based down‐shifting sensitizers and to compare its performance with other low cost methods that have been proposed in the literature.^[^ ^18^ ^]^ One of the key points in our work is to ensure that all the calculations of excited‐state energies are actually corresponding to locally excited states, a question that is often neglected in previous studies where unreliable popular functionals such as B3LYP are used without further consideration. The final goal of developing a protocol for accurate, affordable predictions of the quantum yield will be addressed in the future.

## 2 Computational Details

All density functional theory (DFT)‐based calculations were performed using the ORCA software (version 5.0.3) with a 10^−6^ Eh strict energy criteria and density matrix changes (1 e^−4^ a.u.),^[^ ^19^, ^20^ ^]^ employing Grimme dispersion corrections with Becke–Johnson damping to consider the noncovalent weak interactions between ligands.^[^ ^21^ ^]^ For the forces, the tight self‐consistent field option uses a maximum displacement of 4 e^−3^ Eh bohr^−1^ (Root mean sqaure (RMS), for the displacement of 2 e^−3^ Eh bohr^−1^). In all calculations, Eu^3+^ has been replaced by Y^3+^ (see the discussion of this issue below), and the triple‐ξ valence basis set with polarization functions for all elements (def2‐TZVP) by Ahlrichs and coworkers has been used.^[^ ^22^, ^23^ ^]^ The corresponding vibrational analysis was done for all optimized structures to ensure that they actually correspond to minima on the corresponding potential energy surfaces (*S*_0_ or *T*_1_). In order to assess the performance of DFT‐based methods in the computations of the excited states energies for the ligands, different types of exchange‐correlation (XC) functionals were selected: the perdew‐burke‐erzenhof (PBE) functional,^[^ ^24^ ^]^ which is based on the generalized gradient approximation (GGA) and the most popular hybrid functional, B3LYP.^[^ ^25^ ^]^ Additionally, to properly describe interligand charge‐transfer effects, two long‐range corrected functionals have been used, ωB97X‐D^[^ ^26^ ^]^ and CAM‐B3LYP.^[^ ^27^ ^]^

In principle, one would like to use Eu^3+^ during the geometry optimizations, but its complex electronic structure requires a higher level of theory to account for the multiconfigurational nature of the 4*f* ^6^ configuration. Because we want to reproduce the arrangement of the ligands within the coordination compounds at a reasonable computational cost, the use of DFT‐based methods with some approximations poses as the most reasonable option. All DFT calculations presented in this work were thus performed replacing the Eu^3+^ cation by a Y^3+^ one for both the ground singlet (*S*_0_) and the first excited triplet state (*T*_1_) geometry optimizations. This approximation is based on considering that *f* electrons have a rather small influence on the bonding between the Ln^3+^ cation and the ligands, and also that the absorption and excitation processes between the ground state and the lowest‐lying triplet state are fully localized on the ligands. A viable alternative, based on using f‐in‐core pseudopotentials for the Ln^3+^ cations, which would allow to model the size effect of changing the Ln^3+^ cation, is currently being tested in our group.^[^ ^28^, ^29^ ^]^

For calculations using semiempirical methods, the Lanthanide Luminescence Software Package (LUMPAC)^[^ ^18^ ^]^ has been employed. LUMPAC has implemented the Sparkle model in conjunction with the Molecular Orbital PACkage (MOPAC2016), which was also used to carry out the geometry optimizations.^[^ ^30^, ^31^ ^]^ In the Sparkle model, the Eu^3+^ cation^[^ ^32^ ^]^ is replaced by a Coulombic +3 charge subject to a repulsive potential of generic form as shown in the following equation.

(1) $$V\left(r\right)=e^{-\alpha r}$$

where *α* is a parameter introduced to model the effect of the ion size. This value will change depending on the semiempirical method that is used. As in the case of DFT calculations, different semiempirical methods have been chosen to assess their performance in geometry optimizations. Therefore, combined semiempirical methods with their associated Sparkle model, such as AM1,^[^ ^33^ ^]^ PM3,^[^ ^34^ ^]^ PM6,^[^ ^35^ ^]^ PM7,^[^ ^36^ ^]^ and RM1,^[^ ^37^ ^]^ have been employed.

To assess the effect that different computational approaches (DFT or semiempirical) have on the geometry of the studied systems, different tools have been used to compare the structures, either for the whole complex or just for the first coordination sphere around Eu^3+^. For this purpose, the root‐mean‐square‐deviation (RMSD) has been computed between two given structures,^[^ ^38^, ^39^ ^]^ applying Kabsch's algorithm to calculate the optimal rotation matrix that minimizes the RMSD value.^[^ ^40^ ^]^ Similarly, using the Cosymlib library, we employed continuous shape measures (CShM) to describe the coordination polyhedra around the metal center upon optimization in the different studied systems.^[^ ^41^, ^42^ ^]^

Finally, the energies for the excited states of the ligands in the complexes have been calculated by two different methods: time‐dependent density functional theory (TD‐DFT)^[^ ^43^ ^]^ or the semiempirical intermediate neglect of differential overlap (INDO/S) method^[^ ^37^ ^]^ to obtain the first 20 roots corresponding to either singlet or triplet excited states. Excited state calculations were performed for both the ground singlet and first‐triplet excited‐state geometries. In the following, the reported *T*_1_ energy corresponds to the difference between the TD‐DFT *T*_1_ energy obtained with the triplet geometry and the *S*_0_ energy obtained for the singlet geometry.

In addition, the TheoDORE program was used to quantify the extent and nature of charge transfer in the different complexes.^[^ ^44^ ^]^ This analysis has been applied for both TD‐DFT and INDO/S methods.

Since direct computation of the Eu^3+^‐centered excited states requires strongly time‐consuming computational methods, we have adopted the poor man's solution of using experimental values for these energies determined for the bare Eu^3+^ cation. This approximation is, however, reasonable if one takes into account that the ligand field around a Ln^3+^ ion is only able to induce energy changes in these states of around 300 cm^−1^, thus making the bare ion approximation a reasonable one.^[^ ^45^ ^]^ On the bright side, this approximation allows us to employ the method for any Ln^3+^ cation as long as the geometry optimized for the analogous Y^3+^ complex remains a good approximation to the complex's real geometry.

Energies for the lowest‐singlet excited state (*S*_1_) have also been computed, using the ground‐state geometry in this case. However, due to the limited available experimental data, it has not been possible to compare computed and experimental data with the precision of its triplet equivalent and will only be discussed briefly in the following.

## 3 Results

**Figure 2:**
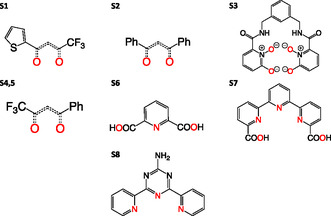
Ligands used for the structural benchmark of Eu^3+^ complexes in this work. The coordination complexes corresponding to ligand **S4,5** are [Eu(**S4**)_3_(H_2_O)_2_] and [Eu(**S5**)_3_(4‐4′‐bpy)(EtOH)]. Nevertheless, the ligand responsible for excitation is the one depicted in the figure. For each ligand, donor atoms directly coordinating to Eu^3+^ are highlighted in red.

The aim of this work is to develop an accurate computational model for the *T*_1_ excited‐state energy calculation in Eu^3+^‐based coordination compounds. By balancing accuracy and computational cost, one should be able to screen across large datasets of complexes searching for potential new candidates that can act as photosensitizers. Ideally, this method must provide accurate coordination geometries that reproduce the geometrical data as well as the energy value of the *T*_1_ excited‐state energy in Eu^3+^ systems. Thus, we recovered a dataset of Eu^3+^‐based complexes that contain both structural and photochemical data (see Table S1, S3, and S8, Supporting Information). In this dataset, only eight compounds with the ligands depicted in **Figure** **2** have crystallographic data for the singlet‐state geometry, due to the difficulty that most of these compounds present toward crystallization. These molecules, though, offer the perfect platform to calibrate computational methods toward accurate calculation of coordination geometries in Eu^3+^‐based complexes.

### 3.1 Geometry Optimizations

To calibrate the performance of the different methods to describe the complexes’ geometries, a series of Eu^3+^ complexes with the ligands depicted in Figure 2 for which accurate structural information is available has been selected (see Table S1, Supporting Information). Eu^3+^ coordination compounds may have different coordination numbers due to their large coordination flexibility. To obtain the most representative results, examples for the most usually observed coordination numbers in these compounds (8, 9, and 10) have been included in the calibration. Also, different ligand denticity has been considered, specifically, tridentate (ML_3_ for coordination number 9) and tetradentate complexes (ML_2_ for coordination number eight). As donor atoms, we only used nitrogen and oxygen, the only ones that appear in the experimentally determined structures.

**Figure 3:**
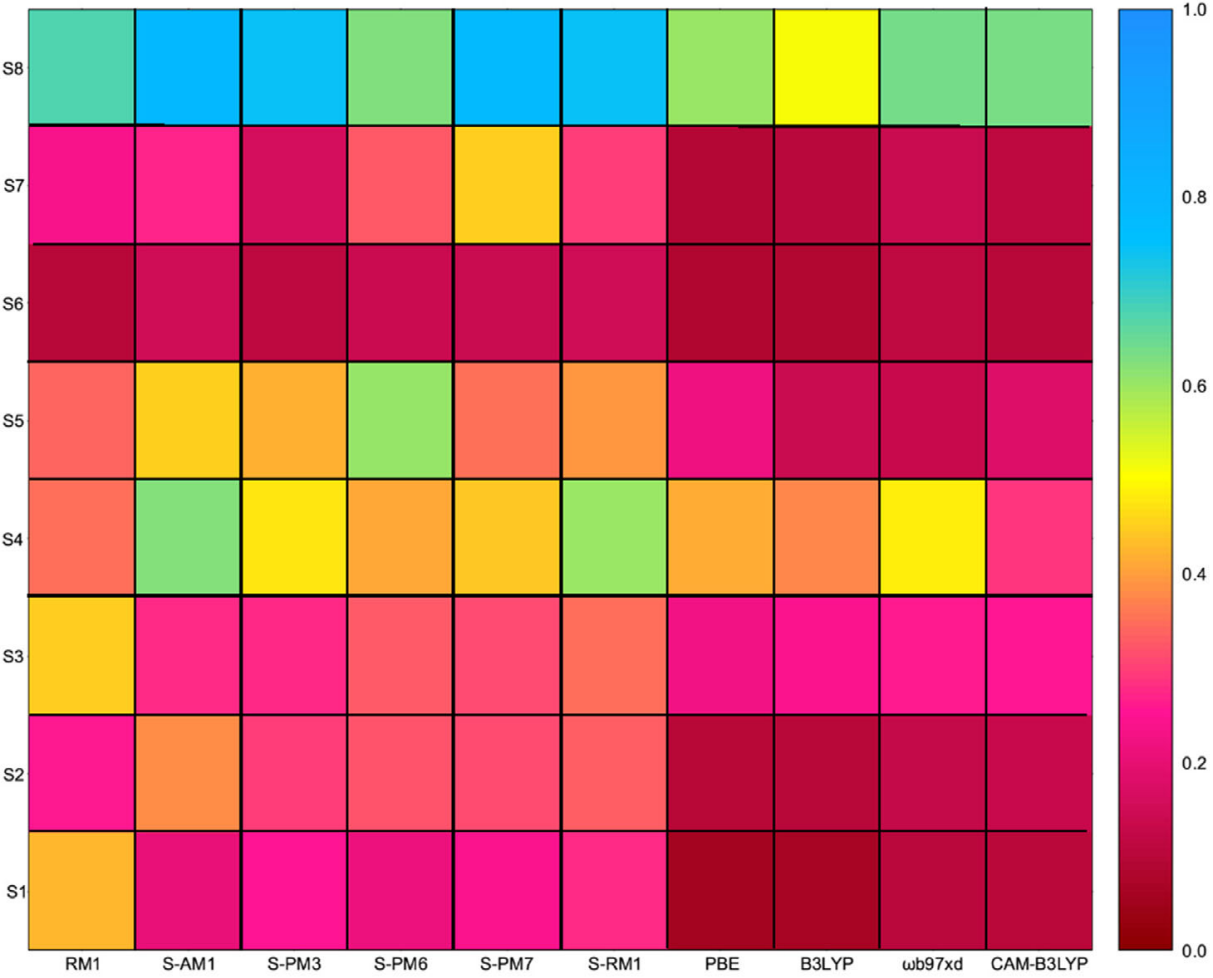
RMSD with respect to the reference experimental structures for the coordination spheres of Eu^3+^ complexes generated with the ligands depicted in Figure 2 using all available computational methods.

Crystallographic data from the Cambridge Structural Database (CSD, v5.43)^[^ ^46^ ^]^ were retrieved for the following compounds (see Table S1, Supporting Information): [Eu(**S1**)_3_(THF)_2_], [Eu(**S2**)_3_(DMA)], [Eu(**S3**)_2_]^−^, [Eu(**S4**)_3_(H_2_O)_2_], [Eu(**S5**)_3_(4‐4′‐bpy)(EtOH)], [Eu(**S6**)_3_]^3−^, [Eu(**S7**)_2_]^−^, and [Eu(**S8**)(NO_3_)_2_(H_2_O)_2_], where **S1** to **S8** refer to the ligands shown in Figure 2. For all such systems, the ground‐state structure was fully optimized using all the semiempirical and DFT‐based methods described in the computational details section. To evaluate the accuracy of such methods toward reproducing the available structural data, the corresponding RMSD values between the optimized and the X‐ray structures (**Figure** **3**) were computed for their first coordination spheres. The RMSD values obtained for the semiempirical methods range between 0.34 and 0.39, while those for the DFT‐based methods range between 0.20 and 0.24, with B3LYP being the functional yielding the lowest value (0.20).

While, in general, structural data are properly reproduced, either by DFT‐based methods or by the semiempirical Sparkle models, from such analysis, it is clear that the B3LYP exchange/correlation functional with the becke‐johnson correction provides a correct balance between accuracy and computational cost and, hence, it was adopted as the reference method for geometry optimizations, hoping that it will generate also reasonable structures for compounds for which the detailed structure is not known. Bond lengths between the metal (Y) and the donor atom obtained in our optimizations are in excellent agreement with the experimentally observed ones (for Eu), according to the statistical data from the CSD (see Supporting Information). It is worth noting that the experimental structure for [Eu(**S8**)(H_2_O)(NO_3_)_2_]^−^ cannot be correctly reproduced with any of the explored methods. This is due to the particular compression that the molecule suffers from its environment within the crystal, an effect that cannot be reproduced by the calculations done here for a single isolated molecule.

**Figure 4:**
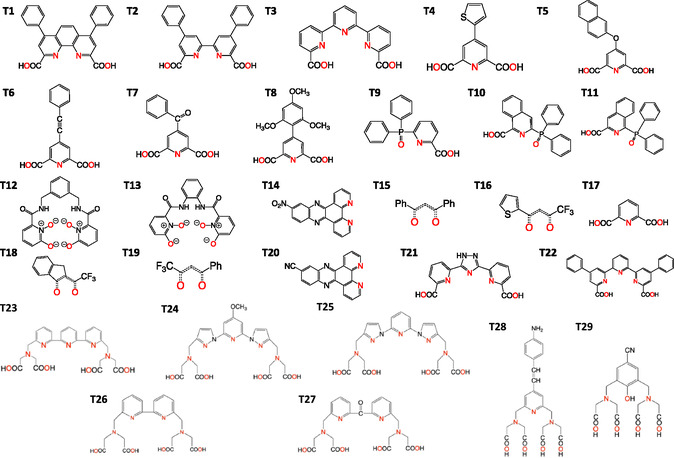
Dataset of ligands in Eu^3+^‐based coordination compounds used in this work for the benchmark calculation of the *T*_1_ state energy. Structural information is also available for compounds with ligands **T3**, **T12**, **T15**, **T16**, **T17**, and **T19**. Donor atoms are highlighted in red.

Many more Eu^3+^ complexes can be found, for which the energy of the *T*_1_ state has been experimentally determined, while no structural information is available for them. Therefore, a dataset of Eu^3+^ coordination compounds for which this value has been measured was assembled (**Figure** **4**). Notice that with only two exceptions ([Eu(**S4**)_3_(H_2_O)_2_] and [Eu(**S8**)(NO_3_)_2_(H_2_O)_2_]), all ligands from Figure 2 appear as well in this group, leading to a total of 29 Eu^3+^‐based coordination compounds, for which the experimental value of the *T*_1_ state is available (see Table S8, Supporting Information). Because for most of the systems in Figure 4, there is no experimental structural data, an *S*_0_ ground‐state geometry for these compounds needs to be modeled. To generate these reference geometries, we will use the B3LYP exchange/correlation method, which, as shown in Figure 3, provides the most accurate geometries when compared with experimental data, thus indicating that it is a reliable method to compute geometries in Eu^3+^‐based complexes. The generated models attempt to achieve the most common coordination number for Eu^3+^ complexes, this is, nine‐coordination. Of course, this is not always achievable. For instance, ligand **T2**, which has hapticity four, was used to build the [Eu(**T2**)_2_]^−^ complex, in which the metal center is eight‐coordinated. In general, efforts were made to generate homoleptic compounds when experimental information was not available. The complete set of structures generated with their stoichiometry can be found in Table S3, Supporting Information.

A set of reference structures for the *S*_0_ ground state was generated for all compounds with the ligands included in Figure 4 using the B3LYP functional. After that, all the methods described in the computational details section were employed to try to reproduce such structures, and the RMSD values for those versus the B3LYP structures were computed. The overall results are summarized in Table S4, Supporting Information.

In general, semiempirical methods provide structures with slightly larger RMSD values than the DFT‐based methods, with a larger dispersion toward the reference structures. If the average RMSD is analyzed for the semiempirical methods, the value ranges between 0.33 and 0.60, while for the DFT‐based methods, it ranges between 0.07 and 0.16. Across such methods, PBE is the one with the largest differences toward the B3LYP optimized structures, while both ωB97X‐D and CAM‐B3LYP provide (with some exceptions) very similar results as the B3LYP method, yet at a higher computational cost. Thus, from the above analysis, it seems that B3LYP is a reasonable method of choice to obtain reliable ground‐state structures at a reasonable computational cost.

As introduced previously, optimizations are also carried out for the triplet geometry. Since there is no structural data available for the complexes in their *T*_1_ excited state, the geometry and coordination sphere for *T*_1_ geometry obtained using B3LYP has been systematically compared with that obtained using the other methods to evaluate the differences that may arise in the reorganization of the coordination complex upon excitation using a different DFT approximations. The RMSD values obtained using semiempirical methods exhibit a considerable increase in their values (average RMSD 0.44), leading to a greater dispersion in the data (see Table S5, Supporting Information). However, for the DFT‐based methods, the range of RMSD values remains consistent with the one obtained for the ground‐state geometries (average values 0.10). The relatively small RMSD values for the coordination sphere of Eu^3+^ between the ground and excited‐state structures indicate that there are no significant structural differences in the first coordination sphere between the two states, as expected if the atomic reorganization only affects the ligand that is being excited and not the entire complex.

### 3.2 Triplet Excitation Energy

Since now we can generate reliable structures for the compounds studied in this work, the next step will be to validate the different methods toward the accurate prediction of the *T*_1_ state energy relative to that of the *S*_0_ ground state. Because the excitation is localized in the ligands, experimental data is often obtained from coordination complexes, in which the central Eu^3+^ ion has been replaced by Gd^3+^. Experimentally, it has been observed that there are no significant geometrical differences between coordination complexes obtained with either Eu^3+^ or Gd^3+^. In our case, and because of the localized nature of the transition, one step further can be taken, replacing Eu^3+^ by Y^3+^ or the Sparkle model, to compute the energy of the *T*_1_ state on the ligands.^[^ ^47^, ^48^, ^49^, ^50^, ^51^ ^]^ As mentioned above, to compute the relative *T*_1_ state energy, two different structures are employed, each optimized forcing singlet and triplet spin multiplicities, respectively. TD‐DFT or INDO/S calculations are then used for these structures, with the energy for the *S*_0_ ground state extracted from the singlet structure calculation and the energy for the *T*_1_ state obtained from the triplet structure. Having optimized singlet and triplet geometries using all methods, the *S*_0_ ‐ *T*_1_ gap has been computed using geometries obtained with different methods. To maintain clarity regarding the calculation method, the notation “geometry optimization method/excited‐states method” is employed. For instance, B3LYP/ωB97X‐D indicates a ωB97X‐D TD‐DFT calculation for a B3LYP optimized geometry.

**Figure 5:**
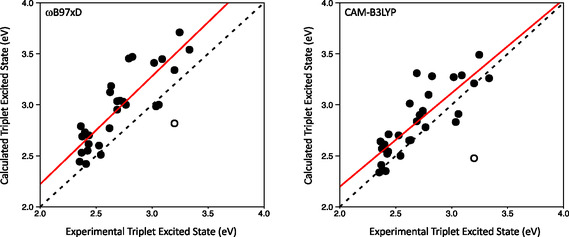
Experimental against computed (left, ωB97X‐D/ωB97X‐D, right,/CAM‐B3LYP CAM‐B3LYP) triplet state energy for coordination complexes with ligands from the T set (Figure 4). The dashed line represents a perfect match between computed and experimental values while the red line corresponds to the linear fit of the computed values. Correlation coefficients for the linear fits are 0.72 for ωB97X‐D and 0.69 for CAM‐B3LYP. The outlier marked with a hollow symbol and corresponding to the **T20** ligand has not been considered in the linear regression. This ligand was discarded because the computed *T*_1_ energy was unreasonably low for this type of chromophore, containing a cyano group, and thus lacked chemical validity.

Both semiempirical and DFT‐based methods were used to compute the energy of the *T*_1_ state. On one hand, none of the semiempirical/INDO/S approaches offers any clear correlation against the experimental data (see Table S7, Supporting Information), which makes us disregard the use of such methods for the calculation of *T*_1_ state energies. When using DFT methods, two trends are observed. The PBE/PBE results, as well as the B3LYP/B3LYP results, do not seem to provide a clear correlation against experimental data and introduce also interligand charge transfer in certain unpredictable cases. Only the ωB97X‐D/ωB97X‐D and CAM‐B3LYP/CAM‐B3LYP results do show a clear correlation against the experimental data and, more importantly, provide a correct description of the excitation's nature, that is, the excitation remains localized on a single ligand for all cases as verified using the TheoDORE program. In **Figure** **5**, we show the correlation between the computed and the experimental values of the *T*_1_ state energy using the ωB97X‐D/ωB97X‐D and CAM‐B3LYP/CAM‐B3LYP methods for Eu^3+^‐based coordination compounds with the ligands of Figure 4. As it can be seen from the figures, CAM‐B3LYP/CAM‐B3LYP offers a slightly better correlation than the ωB97X‐D/ωB97X‐D calculations. While long‐range corrected density functionals provide a systematic overestimation of ≈0.15 eV respect to the experimental values, this overestimation is practically constant in the 2.0–4.0 eV range. Obviously, one would like the method to match the experimental data exactly, but CAM‐B3LYP/CAM‐B3LYP is, across the studied methods, the one that consistently provides the most reliable value for the computed value of the *T*_1_ state across the whole range of measured *T*_1_ energies.

**Figure 6:**
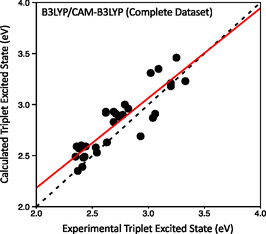
B3LYP/CAM‐B3LYP versus experimental triplet state energies for coordination complexes with ligands from the T set (Figure 4). The dashed line represents a perfect match between computed and experimental values while the red line corresponds to the linear fit of the computed values ($T_{1\left(\text{calc}\text{.}\right)}=0.88\cdot T_{1\left(\text{exp}.\right)}+0.43,R^{2}=0.78)$.

As concluded in the previous section, it was determined that the optimal method for conducting optimizations is B3LYP, while for the TD‐DFT calculation of the *T*_1_ state energy, the CAM‐B3LYP functional is preferred. Thus, an optimal approach for the *T*_1_ state‐energy calculation should be a geometrical optimization with B3LYP, followed by a TD‐DFT calculation with CAM‐B3LYP (B3LYP/CAMB3LYP in our notation). In principle, the energy of the *T*_1_ state could also be obtained from a DFT calculation for the triplet geometry enforcing a 2S+1 = 3 spin multiplicity, thus avoiding the need to use TD‐DFT at all. However, since the outlined protocol requires the change of functional in order to get accurate results for the *T*_1_ state energy, and an additional calculation is necessary in any case, TD‐DFT also allows us to get the energies of the different S_n_ states for the two geometries, a feature that will be helpful for future developments. In **Figure** **6**, we show the results for the B3LYP/CAMB3LYP protocol on our dataset.

Given its overall performance, subsequent calculations were carried out using the B3LYP/CAM‐B3LYP method to compare the obtained results with the experimental data.

### 3.3 Triplet‐Excited‐State Energy Model Development

At this point, the outlined methodology (B3LYP/CAM‐B3LYP) has proven to be able to accurately model the energy of the triplet excited state using electronic structure methods, but with some systematic bias. As can be seen from Figure 5, the CAM‐B3LYP/CAM‐B3LYP method can be fitted using a linear regression model, which in turn should allow us to estimate the actual experimental energy of the triplet excited state, but the linear fit does not exactly overlap with the diagonal, especially for low excitation energies, indicating that some methodological bias is present. Thus, we wondered if it would be possible to build a similar linear model derived from the B3LYP/CAM‐B3LYP results (as in Figure 6) that could, with precision, estimate the experimental value of the triplet excited state. If that is the case, the model should be able to give us the position of the *T*_1_ state, which should match the experimental value.

**Figure 7:**
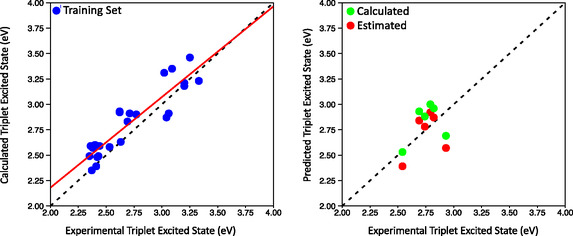
Left, training set used to generate a linear model to calculate the *T*_1_ state energy (B3LYP/CAM‐B3LYP). This linear model can be inverted to predict the experimental *T*_1_ state energy for the compounds in the validation set. Right, comparison of the computed *T*_1_ states (B3LYP/CAM‐B3LYP, in green) versus the predicted *T*_1_ state using the linear model (red).

To develop this model, we need to use the T dataset (a total of 29 data points), and split this group into two new subsets, one containing 80% of the data (training set) and another one containing 20% of the data (validation set), which is randomly selected, to check the accuracy of the model. Using the linear fit from the training set, it is possible to estimate the experimental value for the test set, which we can compare with the actual experimental value for the triplet state (see Jupiter Notebook, Supporting Information). Afterward, this model can be used to predict the *T*_1_ energies of the validation set, which improves the accuracy of the predictive model. Results are shown in **Figure** **7**.

Due to the limited size of our dataset, one may wonder to what extent the selection of the training set will affect the results. To test the consistency of this methodology, random sampling was done following the 80%–20% split between training and validation sets. The validation set is key because we have the actual experimental values of the triplet‐excited‐state energies for such systems, which will allow us to quantify the error that the model has in each run. Each training set was used to build the corresponding linear model for the calculation of the *T*_1_ state (B3LYP/CAM‐B3LYP), which later allowed us to compute the predicted *T*_1_ state energy (B3LYP/CAM‐B3LYP) for each molecule in the validation set, and compare it with the experimental *T*_1_ energy value.

In our particular case, and using software developed by ourselves (see Jupyter Notebook, Supporting Information), this process was performed ten consecutive times, generating every one of them random training (80%) and validation (20%) sets across the complete dataset, which allows us to estimate the confidence interval of the slope and intercept values for the linear model. As can be seen from the data (see Table S12, Supporting Information), the selection of the training set does not introduce significant dispersion in our model. With this approach, the confidence intervals for both the slope and the y‐axis interception value can be estimated ($T_{1\left(\text{calc}\text{.}\right)}=0.864\left(\pm 0.032\right)\cdot T_{1\left(\text{exp}.\right)}-0.470\left(\pm 0.083\right))$, indicating that no major changes are obtained regardless of the training/validation splitting. Indeed, the average error, below 1000 cm^−1^, remains more or less constant through the runs (see Table S12, Supporting Information). The equation obtained to predict the *T*_1_ value from the computed *T*_1_ (B3LYP/CAM‐B3LYP) energies can therefore be outlined as:

(2) $$T_{1\left(\text{pred}.\right)}=1.158\cdot T_{1\left(\text{calc}.\right)}-0.544$$

An attempt to build an analogous model for the *S*_1_ state was attempted, although due to the limited number of reliable experimental data on the position of such state, the validation of the model's predictions becomes quite difficult, and we are not confident enough in proposing a model to accurately predict the *S*_1_ excited‐state energy.

## 4 Discussion

Before going into depth, let us discuss about the reliability and reproducibility of the experimental structural data. Figure 3 illustrates that semiempirical methods often exhibit a significant variability in reproducing both the available experimental structures (dataset S) and the reference structures generated using the B3LYP functional for compounds with ligands of the T set. This is, somehow, expected due to the parameterization of the semiempirical models, which eventually may lead to certain structural features that do not appear when optimizations are done with DFT methods. While indeed, semiempirical methods are quite cheap from the point of view of the computational cost, the nonsystematical variability in their results when analyzing the geometry of the coordination sphere makes us disregard them as a reliable tool to compute structures when these are not experimentally available.

A complete analysis of the coordination environment was done using the CShM methodology to analyze the geometry of the coordination polyhedron for each optimized structure (see S9 section, Supporting Information). The CShM analysis reveals an excellent agreement between the shape of the optimized polyhedra and the experimental ones. A capped octahedron (coordination number 7),^[^ ^52^ ^]^ square antiprism and trigonal dodecahedron (coordination number 8),^[^ ^53^ ^]^ tricapped trigonal prism and muffin geometry (coordination number 9),^[^ ^54^ ^]^ and a bicapped square antiprism (coordination number 10)^[^ ^55^ ^]^ have been found as the most reliable descriptions for the coordination sphere in agreement with the structural trends previously described for such coordination numbers. One must be careful though, because even though matching the coordination sphere is a good indicator, sometimes the shape measure deviates due to compression or elongation of the coordination sphere, thus making the unambiguous assignment difficult. In such cases, the continuous symmetry measures (CSM) can be a complementary descriptor to identify the reference polyhedron because they quantify symmetry elements that persist regardless of such type of distortions.^[^ ^55^ ^]^ Shape measures for the structures optimized using DFT always show a match with the shape of the coordination polyhedron observed in the crystal structure. Obviously, in some cases, there are additional relevant crystal‐packing effects that lead to certain distortions in the crystal structure that cannot be properly reproduced by any method using just optimizations for isolated molecules. On the other hand, structures obtained using semiempirical methods often show a different closest coordination polyhedron than for the experimental crystal structures. Although in some cases, a match between the shape of the coordination polyhedron in the crystal structure and in the optimized structure is observed, in general, no clear trend can be extracted. This fact, in our opinion, makes it difficult to use semiempirical methods as an accurate strategy to optimize unavailable structures.

Nonetheless, the most accurate method we have found to perform geometry optimizations at an affordable computational cost is the use of DFT calculations for the analogous Y^3+^ compound with the def2‐TZVP basis set and the hybrid exchange/correlation B3LYP functional, including Grimme dispersion corrections with Becke–Johnson damping. We compared the overall geometric data produced by the B3LYP optimizations with the available structural data for the S set, which shows an average difference in the metal–ligand bond lengths of only 0.028 Å for the whole set. This value reinforces the approximation that replacement of Eu^3+^ by Y^3+^ in the optimizations does not introduce drastic changes in the coordination geometry. Because B3LYP has been validated to accurately reproduce experimental ground‐state structures, we expect that the same applies to triplet excited‐state geometries. However, this cannot be confirmed as there are no available experimental structures for such spin multiplicity.

Besides geometrical optimization, a crucial issue for the prediction of the excited triplet energies is the choice of the method used to compute the energy for the triplet state. This is especially sensible for TD‐DFT‐based methods, where different exchange correlation functionals may lead to large differences in energy when computing the energy of the *T*_1_ state. In‐depth analysis of our results shows that PBE systematically yields T_1_ energies, which are too low (see Table S8, Supporting Information) due to an overstabilization of interligand charge‐transfer states during the singlet‐triplet excitation; this is, the electrons move from one ligand to another one (LLCT, ligand‐to‐ligand charge transfer) upon excitation. The effect is clearly visible for the *S*_1_ state, which is practically predicted to have the same energy as the *T*_1_ state. The same phenomenon is also observed in calculations with the B3LYP functional, although for that case, the appearance of low‐lying LLCT states is not systematic as for the PBE functional, so that B3LYP becomes unpredictable for estimating excited‐states energies. In particular, ligand‐to‐ligand charge transfer is observed for structures with ligands T3, T7, and T17. Since this problem can only be detected a posteriori, once the TD‐DFT calculation is done, the use of B3LYP to estimate the energy of the triplet is not advised in the development of a prediction protocol meant to be used without supervision in the screening of potential ligands with a good antenna effect. From our benchmark data, it's clear that only the long‐range corrected functionals, CAM‐B3LYP and ωB97X‐D, are capable of localizing the transition in the same ligand for all studied systems. Since the trend in triplet energies obtained with the CAM‐B3LYP functional is slightly better aligned with the experimental data (Figure 5), we have chosen this functional for our protocol.

Because the singlet to triplet excitation is quite localized on one ligand, one might wonder if a simpler calculation for just the isolated ligand would be enough to accurately predict the energy of the *T*_1_ state in the complex. A difficulty that appears in this strategy is the choice of geometry: should one use the relaxed geometry for an isolated ligand, or should one relax first the structure for the whole complex and perform the excited‐state calculation for the isolated ligand using the specific geometry that it adapts in the complex? To that aim, the same computational strategy has been applied to some of the ligands in our dataset (T1, T13, T14, T15, T21), considering both the same structure for the isolated ligand as in the complex or fully relaxing the structure for a single isolated ligand molecule. The results are, however, quite unsatisfactory because, regardless of the geometry used, the computed *T*_1_ energies obtained for the isolated ligands show a considerable dispersion when compared to the experimental *T*_1_ energies for the complexes (Table S11, Supporting Information), making it impossible to use a calculation for just a single ligand to reproduce the experimental *T*_1_ energies. Moreover, complexation to the metal alters the highest occupied molecular orbital–lowest unoccupied molecular orbital. gap in the ligand. This leads to a larger gap in the complex than in the ligand itself, thus misplacing the position of the *T*_1_ states when calculations are done for the isolated ligand. Therefore, we conclude that, unfortunately, it is necessary to perform the calculations for the whole coordination compound to obtain coherent results that match the experimental data. At this point, it must be stressed that experimentally, the measured triplet excitation energy does not correspond to the isolated ligand. In experiments, the *T*_1_ energy is obtained from a phosphorescence spectrum for coordination compounds, in which Eu^3+^ is replaced by Gd^3+^. This replacement is necessary to avoid the efficient energy transfer from the ligand to the metal that results in a practically total quenching of the phosphorescence from the *T*_1_ state in Eu^3+^ complexes. This reinforces the idea that the isolated ligand is not a proper model to study this excitation.

## 5 Conclusions

In this work, a computational protocol based on DFT calculations is introduced to accurately predict the energy of the triplet state (*T*_1_) in antenna ligands for Eu^3+^ coordination compounds. Using a large enough dataset of structures, different semiempirical and DFT‐based methods have been tested to accurately reproduce the experimentally available data. Particular care has been taken to avoid methods that lead to nonsystematic behaviors (for example, using Sparkle models for geometry prediction or using the B3LYP functional to estimate the energy of low‐lying excited states).

For geometry optimizations, the B3LYP functional employing Grimme dispersion corrections with Becke–Johnson damping offers an appropriate balance between computational cost and accuracy, and can be used, in principle, to accurately model the coordination environment in compounds without available structural data. Semiempirical methods, although quite appealing from the point of view of computational cost, give a larger dispersion of results when comparing with experimentally available data, leading occasionally to wrong coordination geometries, thus making them less useful in terms of unsupervised predictions.

Similarly, the long‐range corrected CAM‐B3LYP exchange correlation functional is able to reproduce, with only a slight bias, the experimental value for the *T*_1_ state. This method, which correctly localizes the electronic transition on one ligand in all studied compounds, systematically overestimates the energy of the triplet state, but allows us to build a simple model that can be used for an accurate prediction of the *T*_1_ state energy. In turn, this allows us to check if these values lie within the optimal range needed to detect ligands that can lead to an efficient energy transfer to the low‐lying ^5^D_J_ states of Eu^3+^. Even though the excitation is mainly located on one ligand molecule, from our results, it seems clear that, as it happens in the experimental case, only proper values of the *T*_1_ state energy can be obtained considering the ligand within the whole coordination compound.

Thus, it has been demonstrated that an accurate estimation of the triplet energy state for different antenna ligands with an overall error for the whole process below 10% is possible. This can be achieved by using a combination of geometry optimizations for the lowest singlet (*S*_0_) and triplet (*T*_1_) states of a given compound, where Eu^3+^ has been replaced by Y^3+^ using the B3LYP functional with a standard triple‐ξ plus polarization basis set, followed by a TD‐DFT calculation of the excitation energies using the CAM‐B3LYP functional. The *T*_1_ energy (relative to the *S*_0_ energy) can then be used to predict the triplet energy with errors below 1000 cm^−1^ = 0.12 eV with respect to the known experimental values, a precision that is in most cases sufficient to detect if the ligand's triplet state is well aligned with the low‐lying states of the lanthanide cation (Eu^3+^ in our case). In this sense, our protocol can be safely used in high throughput searches for ligands with an efficient antenna effect. Note, that the alignment of the *T*_1_ energy with the low‐lying excited states of the lanthanide cation is just a necessary, but not sufficient condition for a good quantum yield for the antenna effect and further work is still needed to develop a reliable method to predict novel organo‐lanthanide spectral converters with high quantum yields.

## Conflict of Interest

The authors declare no conflict of interest.

## Supporting information

Supplementary Material

## Data Availability

The data that support the findings of this study are available in the supplementary material of this article.
