# Supplementary material for: Computational Strategies for Predicting Excited‐State Energies in Eu3+ Down‐Shifting Spectral Converters for Photovoltaic Devices
Source: Chemphyschem. 2025 Nov 23;27(4):e202500543. doi: 10.1002/cphc.202500543 (PMC12931575; doi:10.1002/cphc.202500543)

Supporting Information

Computational strategies for predicting excited state energies in Eu^{3+} down-shifting spectral converters for photovoltaic devices

Laura Sanchez-Muñoz[1], Daniel Aravena[2], Jordi Cirera[3] and Pere Alemany[1]**

[1] Departament de Ciència de Materials i Química Física and Institut de Recerca de Química Teòrica i Computacional, Universitat de Barcelona (IQTC-UB), Diagonal 645, 08028 Barcelona, Spain

[2] Laboratory of Computational Inorganic Chemistry, Faculty of Chemistry and Biology, Universidad de Santiago de Chile, Avenida Libertador Bernardo O'Higgins 3363, Estación Central, Santiago, Chile

[3] Departament de Química Inorgànica i Orgànica and Institut de Recerca de Química Teòrica i Computacional, Universitat de Barcelona (IQTC-UB), Diagonal 645, 08028 Barcelona, Spain

e-mail: p.alemany@ub.edu

e-mail: jordi.cirera@qi.ub.es

S1 Crystallographic data retrieved from the Cambridge Structural Database (CSD, v5.43) 3

S2 Root Mean Square Deviation Coordination Environment 4

S3 Dataset ligands for the benchmark calculation of the T_1 state energy 5

S4 Root Mean Square Deviation Coordination Environment (T_1 dataset, singlet) 6

S5 Root Mean Square Deviation Coordination Environment (T_1 dataset, triplet) 7

S6 Root Mean Square Deviation Coordination Environment Figure (T_1, S) 8

S7 T_1 Calculated energies using semiempirical methods 9

S8 T_1 Calculated Energies using TD-DFT 10

S9 Continuous Shape Measures for S dataset 11-14

S10 Average distances Metal-Ligand for experimental and optimized structures 15

S11 T_1 energies for isolated ligands 16

S12 Regression models generated from the overall dataset 17-18

S13 Cartesian coordinates and output calculations additional data 19

S14 Statistical treatment of M-O and M-N bonds from structural data 20

S1 Crystallographic data retrieved from the Cambridge Structural Database (CSD, v5.43)

Ligand	Stoichiometry	Name	Refcode
S1	$[Eu(S1)_3(THF)_2]$	1,1,1-trifluoro-4-oxy-4-(thiophen-2-yl)but-3-en-2onato	FAMNAM
S2	$[Eu(S2)_3(DMA)]$	Dibenzoylmethanato	RATKUU
S4	$[Eu(S4)_3(H2O)_2]$	Benzoyl-1,1,1-trifluoroacetonato	TOGHUS
S5	$[Eu(S5)_3(4\text{-}4'\text{-}bpy)(EtOH)]$	4,4,4-trifluoro-1-phenyl-1,3-butanedione	GIPCAK
S6	$[Eu(S6)_3]^{3-}$	Dipicolinic acid	COYCEA
S7	$[Eu(S7)_2]^{-}$	2,2':6',2"-Terpyridine-6,6"-dicarboxilic acid	YUFFAH
S8	$[Eu(S8)(NO_3)_2(H_2O)_2]$	4-Amino-2,6-bis(2-pyridil)-1,3,5-triazine	DUCNAQ
T18	$[Eu(T18)_3(H_2O)_2]$	2-(trifluoroacetyl)-1H-inden-3-olato	GAWWAE

S2 Root Mean Square Deviation (RMSD) with respect to experimental structures for the coordination environment of Eu^{3+} in the structural dataset **S1 – S8**.

Ligand	RM1	S-AM1	S-PM3	S-PM6	S-PM7	S-RM1	PBE	B3LYP	ωb97xd	CAM B3LYP
**S1**	0.423	0.250	0.212	0.242	0.202	0.272	0.056	0.060	0.102	0.101
**S2**	0.255	0.294	0.317	0.309	0.379	0.330	0.095	0.097	0.123	0.131
**S3**	0.446	0.271	0.325	0.308	0.273	0.345	0.223	0.239	0.257	0.252
**S4**	0.347	0.473	0.407	0.440	0.621	0.599	0.412	0.372	0.481	0.285
**S5**	0.336	0.416	0.603	0.348	0.449	0.389	0.218	0.134	0.131	0.175
**S6**	0.097	0.108	0.136	0.134	0.147	0.146	0.081	0.082	0.110	0.097
**S7**	0.237	0.159	0.323	0.448	0.267	0.295	0.091	0.100	0.134	0.109
**S8**	0.672	0.741	0.624	0.779	0.785	0.742	0.600	0.507	0.635	0.631

S3 Ligands and coordination compounds included in the dataset for the benchmark calculations of the T_1 state energy.

Ligand	Complex	Name
**T1**	$[M(Ln)_2]^-$	4,7-diphenyl-1,10-phenanthroline-2,9-dicarboxylic acid
**T2**	$[M(Ln)_2]^-$	6(6-carboxy-4-phenylpyridin-2-yl)-4-phenylpyridine-2-carboxylic acid
**T3**	$[M(Ln)_2]^-$	2,2':6',2"-Terpyridine-6,6"-dicarboxilic acid
**T4**	$[M(Ln)_3]^{3-}$	4-(2-Thienyl)-2,6-pyridinedicarboxilic acid
**T5**	$[M(Ln)_3]^{3-}$	4-(2-Naphthyloxy)-2,6-pyridinedicarboxylic acid
**T6**	$[M(Ln)_3]^{3-}$	4-(Phenylethynyl)-2,6-pyridinedicarboxylic acid
**T7**	$[M(Ln)_3]^{3-}$	4-Benzoyl-2,6-pyridinedicarboxilic acid
**T8**	$[M(Ln)_3]^{3-}$	4-(2,4,6-Trimethoxyphenyl)-2,6-pyridinedicarboxilic acid
**T9**	$[M(Ln)_3]$	6-(diphenylphosphoroso)pyridine-2-carboxylic acid
**T10**	$[M(Ln)_3]$	3-(diphenylphosphoryl)isoquinoline-1-carboxylic acid
**T11**	$[M(Ln)_3]$	1-diphenylphosphoryl-3-carboxyisoquinoline
**T12**	$[M(Ln)_2]^-$	N-[[3-[[(1,6-dioxopyridin-1-ium-2-carbonyl)amino]methyl]phenyl]methyl]-1-oxido-6-oxopyridine-2-carboxamide
**T13**	$[M(Ln)_2]^-$	N,N'-(1,2-phenylene)bis(1-hydroxy-6-oxo-1,6-dihydropyridine-2-carboxamide)
**T14**	$[M(NO_3)_3(Ln)]$	11-nitrodipyrido[3,2-a:20,30-c]phenazine)
**T15**	$[M(Ln)_3(DMA)]$	Dibenzoylmethanato
**T16**	$[M(Ln)_3(THF)_2]$	1,1,1-trifluoro-4-oxy-4-(thiophen-2-yl)but-3-en-2onato
**T17**	$[M(Ln)_3]^{3-}$	Dipicolinic acid
**T18**	$[M(Ln)_3(H_2O)_2]$	2-(trifluoroacetyl)-1H-inden-3-olato
**T19**	$[M(Ln)_3(bpy)(EtOH)]$	(4,4'-bipyridine)-ethanol-tris(4,4,4-trifluoro-1-phenyl-1,3-butanedione)
**T20**	$[M(Ln)(NO_3)_3]$	dibenzo[a,c]phenazine-11-carbonitrile
**T21**	$[M(Ln)_2]^-$	6,6'-(1H-1,2,4-triazole-3,5-diyl)dipicolinic acid
**T22**	$[M(Ln)_2]^-$	4,4"-diphenyl-[2,2':6',2"-terpyridine]-6,6"-dicarboxylic acid
**T23**	$[M(Ln)]^-$	2,2',2",2'"-(([2,2':6',2"-terpyridine]-6,6"diylbis(methylene))bis(azanetriyl))tetraacetic acid
**T24**	$[M(Ln)]^-$	2,2',2",2'"-((((4-methoxypyridine-2,6-diyl)bis(1*H*-pyrazole-1,3-diyl))bis(methylene))bis(azanetriyl))tetraacetic acid
**T25**	$[M(Ln)]^-$	2,2',2",2'"-(((pyridine-2,6-diylbis(1*H*-pyrazole-1,3-diyl))bis(methylene))bis(azanetriyl))tetraacetic acid
**T26**	$[M(Ln)]^-$	2,2',2",2'"-(([2,2'-bipyridine]-6,6'-diylbis(methylene))bis(azanetriyl))tetraacetic acid
**T27**	$[M(Ln)]^-$	2,2',2",2'"-(((carbonylbis(pyridine-6,2-diyl))bis(methylene))bis(azanetriyl))tetraacetic acid
**T28**	$[M(Ln)]^-$	2,2',2",2'"-(((4-(4-aminostyryl)pyridine-2,6-diyl)bis(methylene))bis(azanetriyl))tetraacetic acid
**T29**	$[M(Ln)]^-$	2,2',2",2'"-(((5-cyano-2-hydroxy-1,3-phenylene)bis(methylene))bis(azanetriyl))tetraacetic acid

S4 Root Mean Square Deviation (RMSD) with respect to the B3LYP structures for the coordination environment of Eu^{3+} in the S_0 ground state of compounds in the T dataset.

Ligand	RM1	S-AM1	S-PM3	S-PM6	S-PM7	S-RM1	PBE	ωB97X-d	CAM-B3LYP
**T1**	0.927	1.077	0.331	0.513	1.331	0.865	0.681	0.227	0.020
**T2**	0.639	0.099	0.119	0.143	0.126	0.175	0.357	0.035	0.621
**T3**	0.274	0.183	0.321	0.442	0.257	0.289	0.026	0.074	0.018
**T4**	0.128	0.433	0.456	0.450	0.648	0.461	0.119	0.036	0.030
**T5**	0.099	0.421	0.166	0.450	0.454	0.180	0.084	0.118	0.085
**T6**	0.108	0.803	0.659	0.818	0.768	0.820	0.108	0.143	0.039
**T7**	0.117	0.128	0.168	0.177	0.224	0.155	0.080	0.028	0.023
**T8**	0.117	0.148	0.179	0.172	0.171	0.176	0.069	0.107	0.109
**T9**	0.211	0.161	0.174	0.186	1.355	0.210	0.052	0.034	0.048
**T10**	0.219	0.184	0.162	0.219	1.010	0.254	0.090	0.033	0.022
**T11**	0.210	0.162	0.166	0.203	1.450	0.219	0.103	0.039	0.093
**T12**	0.491	0.285	0.359	0.254	0.229	0.380	0.092	0.041	0.031
**T13**	1.289	1.100	1.266	1.088	1.094	1.276	0.157	0.024	0.029
**T14**	0.353	0.530	0.920	0.827	0.851	0.888	0.845	0.033	0.029
**T15**	0.274	0.293	0.344	0.328	0.382	0.344	0.069	0.144	0.117
**T16**	0.420	0.252	0.237	0.260	0.211	0.292	0.046	0.064	0.069
**T17**	0.074	0.101	0.142	0.132	0.136	0.150	0.059	0.054	0.039
**T18**	0.217	0.359	0.628	0.387	0.459	0.371	0.234	0.190	0.259
**T19**	0.311	0.383	0.624	0.345	0.466	0.402	0.150	0.107	0.152
**T20**	0.353	0.530	0.920	0.827	0.851	0.888	0.845	0.033	0.029
**T21**	0.185	0.440	0.260	0.311	0.538	0.466	0.026	0.061	0.036
**T22**	0.398	0.234	0.376	0.431	0.250	0.349	0.062	0.038	0.021
**T23**	0.156	0.162	0.196	0.212	0.175	0.182	0.029	0.039	0.021
**T24**	0.078	0.132	0.171	0.198	0.190	0.204	0.040	0.046	0.043
**T25**	0.072	0.133	0.170	0.194	0.406	0.202	0.019	0.029	0.015
**T26**	0.311	0.373	0.333	0.329	0.420	0.370	0.010	0.020	0.009
**T27**	1.169	1.140	1.952	1.925	1.947	1.573	0.022	0.040	0.027
**T28**	0.284	0.318	0.316	0.365	0.366	0.471	0.092	0.105	0.091
**T29**	0.195	0.936	0.447	0.482	0.821	0.786	0.136	0.148	0.137

S5 Root Mean Square Deviation (RMSD) with respect to the B3LYP structures for the coordination environment of Eu^{3+} in the T_1 excited state of compounds in the T dataset.

Ligand	RM1	S-AM1	S-PM3	S-PM6	S-PM7	S-RM1	PBE	ωB97X-d	CAM-B3LYP
**T1**	1.132	1.212	1.130	1.244	1.239	1.251	0.719	1.449	0.689
**T2**	1.393	0.313	0.576	1.180	1.195	0.739	0.668	0.161	0.466
**T3**	0.297	0.172	0.267	0.415	0.250	0.269	0.103	0.073	0.074
**T4**	0.445	0.458	0.460	0.725	0.755	0.472	0.133	0.027	0.023
**T5**	0.118	0.411	0.167	0.486	0.565	0.437	0.097	0.139	0.203
**T6**	0.553	0.917	0.852	0.877	0.873	0.564	0.130	0.046	0.029
**T7**	0.129	0.185	0.130	0.182	0.178	0.403	0.095	0.038	0.030
**T8**	0.114	0.205	0.130	0.168	0.148	0.168	0.065	0.143	0.183
**T9**	0.215	0.216	0.187	0.192	0.226	1.783	0.098	0.092	0.107
**T10**	0.216	0.258	0.187	0.164	0.236	1.537	0.098	0.039	0.031
**T11**	0.208	0.239	0.174	0.166	0.200	1.479	0.129	0.110	0.101
**T12**	0.732	0.381	0.293	0.380	0.271	0.237	0.068	0.197	0.182
**T13**	0.902	1.287	1.057	1.240	1.065	1.052	0.109	0.141	0.084
**T14**	0.354	0.529	0.922	0.821	0.848	0.889	0.846	0.318	0.029
**T15**	0.302	0.316	0.377	0.366	0.397	0.371	0.089	0.353	0.141
**T16**	0.742	0.282	0.287	0.844	0.569	0.644	0.133	0.105	0.099
**T17**	0.182	0.216	1.950	0.210	0.210	0.231	0.175	0.229	0.208
**T18**	0.569	0.412	0.435	0.536	0.544	0.502	0.514	0.206	0.536
**T19**	0.327	0.363	0.745	0.705	0.480	0.639	0.375	0.137	0.534
**T20**	0.235	0.341	0.671	0.616	0.640	0.657	0.894	0.351	0.833
**T21**	0.221	0.538	0.249	0.334	0.676	0.509	0.092	0.345	0.053
**T22**	0.531	0.338	0.374	0.450	0.286	0.347	0.170	0.053	0.301
**T23**	0.199	0.211	0.254	0.249	0.646	0.200	0.093	0.109	0.068
**T24**	0.143	0.156	0.193	0.249	0.224	0.237	0.227	0.057	0.045
**T25**	0.122	0.405	0.188	0.231	1.331	0.555	0.086	0.037	0.032
**T26**	0.334	0.317	0.322	0.294	0.331	0.345	0.071	0.034	0.023
**T27**	1.995	1.545	1.858	1.835	0.336	1.846	0.031	0.043	0.031
**T28**	0.281	0.464	0.497	0.366	0.371	0.467	0.072	0.049	0.023
**T29**	0.992	1.091	1.020	1.079	1.042	0.912	0.024	0.055	0.010

S6 Root Mean Square Deviation (RMSD) with respect to the B3LYP structures for the coordination environment of Eu^{3+} in the S_0 ground state of compounds in the T dataset.

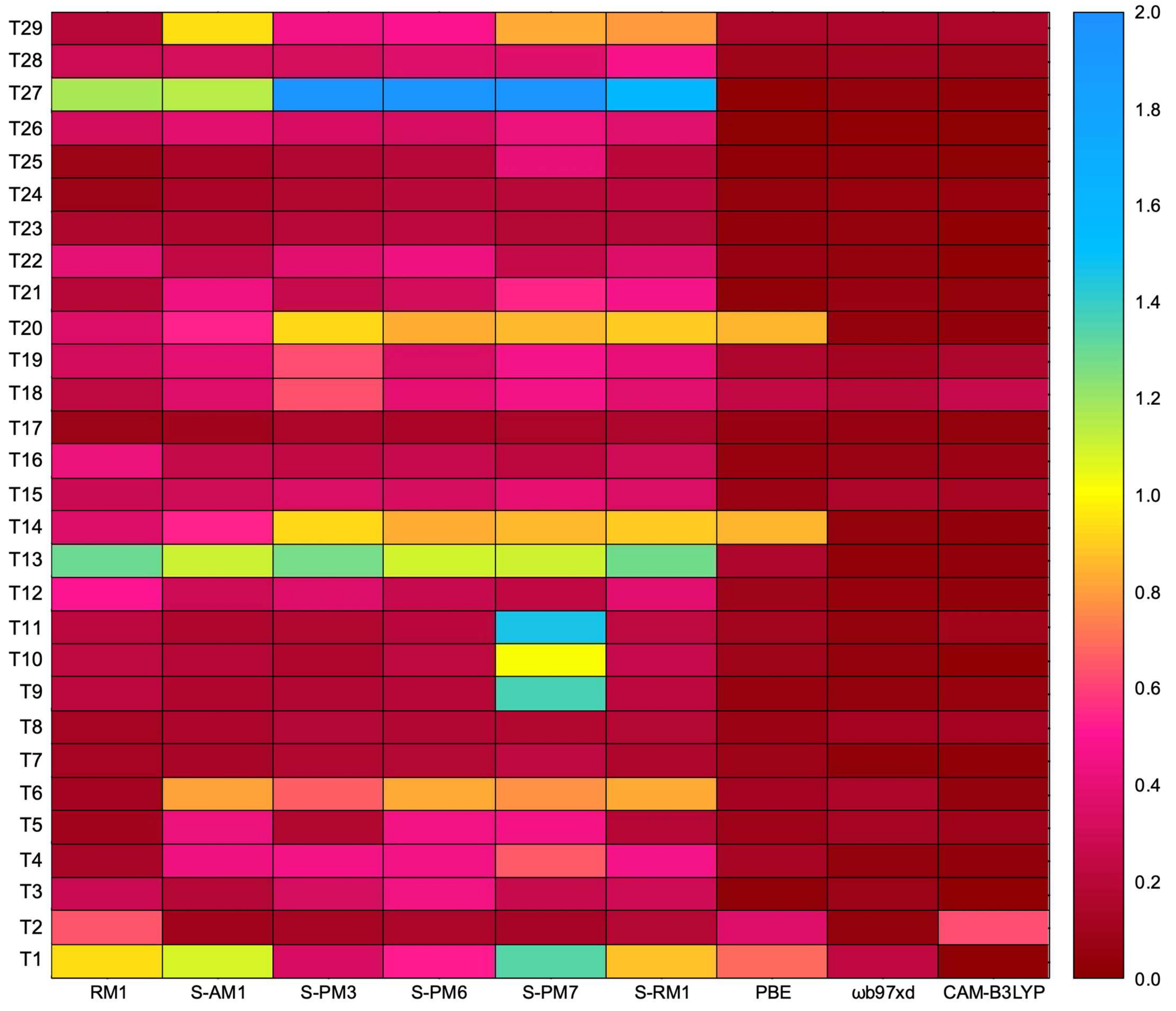

S7 T_1 calculated energies (eV) using semiempirical methods

Ligand	RM1 (eV)	S-RM1 (eV)	S-AM1 (eV)	S-PM3 (eV)	S-PM6 (eV)	S-PM7 (eV)	Exp (eV)
**T1**	2.658	2.521	2.185	2.012	2.176	2.357	2.364
**T2**	2.662	2.613	2.487	2.403	2.595	2.650	2.766
**T3**	2.659	2.632	2.586	2.652	2.536	2.619	2.742
**T4**	2.435	2.139	1.918	1.843	2.119	2.062	2.376
**T5**	2.973	2.771	2.536	2.517	2.706	2.508	2.526
**T6**	3.082	2.703	2.478	2.616	2.597	2.598	2.620
**T7**	3.068	2.995	2.900	3.197	3.181	3.156	2.714
**T8**	3.239	3.089	3.099	2.960	3.101	3.206	2.824
**T9**	3.060	3.009	2.804	3.019	2.821	2.793	3.334
**T10**	2.721	2.712	2.568	2.784	2.734	2.058	2.400
**T11**	2.443	2.455	2.430	2.609	2.632	2.615	2.424
**T12**	2.147	2.087	2.017	2.118	2.094	2.111	2.410
**T13**	2.300	2.145	2.049	2.107	2.048	2.114	2.371
**T14**	2.051	2.048	1.921	1.922	1.998	1.988	3.200
**T15**	2.519	2.544	2.367	2.289	2.388	2.453	2.542
**T16**	1.614	1.675	1.538	1.629	1.688	1.633	2.436
**T17**	3.128	3.136	3.065	3.160	3.111	3.112	3.246
**T18**	2.095	2.087	2.013	1.976	2.069	2.090	2.353
**T19**	2.396	2.418	2.316	2.325	2.340	2.418	2.433
**T20**	2.051	2.048	1.921	1.922	1.998	1.988	3.200
**T21**	2.447	2.285	2.297	2.516	2.363	2.395	2.793
**T22**	2.839	2.839	2.720	2.827	2.738	2.611	2.625
**T23**	2.509	2.392	2.362	2.346	2.363	2.541	2.688
**T24**	2.476	2.359	2.354	2.286	2.232	2.235	3.090
**T25**	2.375	2.297	2.243	2.277	2.202	2.231	3.018
**T26**	2.356	2.420	2.312	2.311	2.375	2.392	2.688
**T27**	2.753	2.749	2.609	2.731	2.691	2.740	3.036
**T28**	2.333	2.341	2.285	2.379	2.171	2.259	2.634
**T29**	2.116	2.149	2.139	1.914	1.861	2.121	3.061

S8 T_1 Calculated Energies (eV) using TD-DFT methods

Ligand	PBE (eV)	B3LYP (eV)	ωB97X-D (eV)	CAM-B3LYP (eV)	Exp (eV)
**T1**	1.569	2.221	2.788	2.643	2.364
**T2**	1.500	2.586	2.995	2.784	2.766
**T3**	1.837	2.724	3.035	2.938	2.742
**T4**	1.117	2.424	2.688	2.573	2.376
**T5**	0.334	2.141	2.598	2.697	2.526
**T6**	0.539	1.854	2.772	2.646	2.620
**T7**	0.303	2.836	3.036	2.902	2.714
**T8**	1.381	2.803	3.467	3.282	2.824
**T9**	1.818	2.558	3.543	3.264	3.334
**T10**	1.777	2.497	2.729	2.610	2.400
**T11**	1.373	2.395	2.547	2.518	2.424
**T12**	2.053	2.235	2.424	2.349	2.410
**T13**	2.034	2.135	2.533	2.410	2.371
**T14**	1.786	2.801	3.338	3.212	3.200
**T15**	2.142	2.360	2.509	2.502	2.542
**T16**	2.452	2.428	2.700	2.201	2.436
**T17**	2.389	2.758	3.710	3.485	3.246
**T18**	2.148	2.245	2.442	2.337	2.353
**T19**	2.042	2.352	2.615	2.542	2.433
**T20**	2.466	2.707	2.818	2.476	3.200
**T21**	1.901	2.854	3.454	3.098	2.793
**T22**	1.603	2.770	3.124	3.012	2.625
**T23**	1.983	2.022	3.035	3.310	2.688
**T24**	2.174	2.510	3.448	3.289	3.090
**T25**	1.132	2.578	3.411	3.271	3.018
**T26**	2.253	2.600	2.952	2.838	2.688
**T27**	1.899	1.969	2.984	2.831	3.036
**T28**	2.634	2.416	3.184	2.654	2.634
**T29**	2.670	2.460	3.000	2.910	3.061

S9 Continuous Shape Measures (CShM) for the coordination environment of Eu^{3+} in the compounds of the structural dataset **S1** – **S8**. The smallest CShM value giving the best description of the coordination polyhedron is highlighted in boldface.

**S1-(ML_8)**			
	**SAPR-8**	**TDD-8**	**BTPR-8**
**Experimental**	**0.217**	2.547	2.173
**PBE**	**0.211**	2.391	1.870
**B3LYP**	**0.229**	2.229	2.040
**ωB97X-D**	**0.442**	1.963	1.503
**CAM-B3LYP**	**0.474**	1.848	1.557
**S-RM1**	3.611	**1.682**	2.365
**RM1**	**1.796**	2.489	3.182
**S-AM1**	**1.593**	1.716	2.193
**S-PM3**	**1.015**	2.553	2.392
**S-PM6**	**1.524**	2.076	2.946
**S-PM7**	**1.083**	2.029	2.322

**S2-(ML_7)**		
	**COC-7**	**CTPR-7**
**Experimental**	**0.529**	1.905
**PBE**	**0.567**	1.357
**B3LYP**	**0.521**	1.143
**ωB97X-D**	**0.805**	1.317
**CAM-B3LYP**	**1.053**	1.732
**S-RM1**	**2.441**	2.658
**RM1**	3.066	2.**937**
**S-AM1**	**2.722**	2.817
**S-PM3**	2.725	1.**910**
**S-PM6**	3.280	3.**224**
**S-PM7**	**4.563**	4.755

S3-(ML_8)		
	**TDD-8**	**BTPR-8**
**Experimental**	**2.184**	2.708
**PBE**	**2.269**	3.572
**B3LYP**	**2.630**	3.845
**ωB97X-D**	**2.078**	3.419
**CAM-B3LYP**	**2.406**	3.678
**S-RM1**	**4.799**	7.259
**RM1**	**4.583**	5.548
**S-AM1**	**4.768**	5.470
**S-PM3**	**4.947**	5.430
**S-PM6**	**5.254**	6.116
**S-PM7**	**4.419**	5.028

S4-(ML_8)				
	**SAPR-8**	**TDD-8**	**JBTPR-8**	**BTPR-8**
**Experimental**	2.958	**0.193**	3.410	3.031
**PBE**	**0.913**	2.787	3.142	2.710
**B3LYP**	**0.615**	2.058	2.747	2.066
**ωB97X-D**	**0.968**	1.797	2.351	1.820
**CAM-B3LYP**	1.833	1.604	1.804	**1.264**
**S-RM1**	2.955	2.245	2.650	**1.931**
**RM1**	**4.565**	4.581	5.759	5.401
**S-AM1**	4.736	**4.723**	5.685	4.964
**S-PM3**	2.721	3.210	3.333	**2.451**
**S-PM6**	5.636	**3.326**	5.520	4.813
**S-PM7**	8.438	6.693	**6.399**	8.322

S5-(ML_8)			
	**SAPR-8**	**TDD-8**	**BTPR-8**
**Experimental**	3.076	**0.420**	2.354
**PBE**	2.703	**0.425**	2.229
**B3LYP**	1.231	**0.640**	1.578
**ωB97X-D**	2.835	**0.646**	1.789
**CAM-B3LYP**	2.036	**0.652**	2.244
**S-RM1**	1.721	2.109	**1.663**
**RM1**	4.019	3.678	**3.361**
**S-AM1**	**2.349**	2.746	2.850
**S-PM3**	2.597	**1.704**	2.017
**S-PM6**	3.004	**2.823**	2.856
**S-PM7**	4.011	**1.840**	3.164

S6-(ML9)				
	**CSAPR-9**	**JTCTPR-9**	**TCTPR-9**	**MFF-9**
**Experimental**	1.534	1.893	**1.222**	2.148
**PBE**	**1.263**	1.614	1.388	1.919
**B3LYP**	1.523	1.586	**1.216**	2.161
**ωB97X-D**	**0.992**	1.771	1.460	1.673
**CAM-B3LYP**	**1.145**	1.707	1.515	1.810
**S-RM1**	1.342	1.582	**0.978**	2.010
**RM1**	2.665	**2.229**	2.584	3.168
**S-AM1**	2.237	**1.836**	2.087	2.792
**S-PM3**	2.576	**2.176**	2.455	3.090
**S-PM6**	2.584	**2.269**	2.513	3.086
**S-PM7**	2.680	**1.927**	2.665	3.198

S7-(ML_{10})				
	**JBCSAPR-10**	**JSPC-10**	**SDD-10**	**HD-10**
**Experimental**	**3.227**	4.411	4.030	3.422
**PBE**	**3.047**	4.535	4.688	3.373
**B3LYP**	**2.856**	4.357	4.67	3.503
**ωB97X-D**	**2.676**	4.094	4.457	3.865
**CAM-B3LYP**	**3.119**	4.727	4.634	3.215
**S-RM1**	4.776	5.410	3.221	**2.070**
**RM1**	6.582	7.826	3.806	**0.76**
**S-AM1**	3.615	4.739	3.890	**2.826**
**S-PM3**	6.703	7.976	3.803	**0.658**
**S-PM6**	8.910	10.243	4.537	**0.478**
**S-PM7**	5.903	7.257	4.167	**1.608**

S8-(ML_9)			
	**CSAPR-9**	**TCTPR-9**	**MFF-9**
**Experimental**	2.013	2.259	**2.000**
**PBE**	**2.016**	2.186	2.336
**B3LYP**	**1.889**	2.334	2.184
**ωB97X-D**	2.298	2.728	**2.161**
**CAM-B3LYP**	2.241	2.727	**2.114**
**S-RM1**	2.837	3.371	**2.813**
**RM1**	4.491	**4.208**	4.240
**S-AM1**	**2.967**	3.768	3.663
**S-PM3**	**2.878**	3.422	2.997
**S-PM6**	**5.430**	5.482	5.544
**S-PM7**	10.517	11.232	**8.993**

S10 Average Metal-Ligand distances (in Å) for the experimental and optimized structures in the structural dataset

Ligand	Exp.	RM1	SAM1	S-PM3	S-PM6	S-PM7	S-RM1	PBE	B3LYP	ωB97X-D	CAM-B3LYP
**S1**	2.420	2.415	2.404	2.472	2.436	2.403	2.472	2.364	2.385	2.352	2.351
**S2**	2.374	2.357	2.376	2.445	2.415	2.366	2.439	2.306	2.304	2.297	2.298
**S3**	2.370	2.436	2.433	2.483	2.453	2.436	2.496	2.365	2.364	2.358	2.355
**S4**	2.410	2.394	2.391	2.463	2.421	2.539	2.466	2.363	2.408	2.357	2.354
**S5**	2.470	2.410	2.402	2.475	2.432	2.416	2.473	2.380	2.449	2.372	2.375
**S6**	2.489	2.471	2.471	2.516	2.474	2.477	2.521	2.467	2.479	2.451	2.453
**S7**	2.556	2.545	2.510	2.538	2.491	2.511	2.548	2.556	2.519	2.542	2.560
**S8**	2.510	2.479	2.409	2.460	2.456	2.506	2.503	2.451	2.466	2.439	2.442

S11 T_1 energies (eV). Experimental values obtained for analogous Gd^{3+} complexes compared to those calculated for the isolated ligands (B3LYP/CAM-B3LYP)

**Ligand**	**Exp. (eV)**	**Calc. (eV)**
**T1**	2.36	2.85
**T2**	2.77	3.15
**T3**	2.74	3.27
**T4**	2.38	2.93
**T5**	2.53	2.85
**T6**	2.62	2.55
**T7**	2.71	2.47
**T8**	2.82	3.37
**T13**	2.45	1.96
**T20**	3.08	2.62
**T21**	2.89	3.51
**T22**	2.71	2.57

S12 Regression lines, experimental and predicted value for the test set and Mean Average Error (MAE) for the 10 randomly generated models. For each run, the dataset was split randomly in 80% training (to build the linear model) and 20% test (6 entries).

Fit # 1: Slope m = 0.892 Intercept b = 0.396 eV R2 = 0.830
[2.74], [[2.6278]
[2.93], [2.8408]
[2.54], [2.4036]
[2.79], [2.6838]
[2.69] ,[2.5718]
[2.82], [2.7175]

MAE = [0.1108]

Fit # 2: Slope m = 0.914 Intercept b = 0.334 eV R2 = 0.817
[2.36], [[2.2175]
[2.35], [2.2065]
[2.69], [2.5787]
[2.53], [2.4036]
[2.93], [2.8414]
[2.79], [2.6882]]

MAE = [0.119]

Fit # 3: Slope m = 0.835 Intercept b = 0.536 eV R2 = 0.770
[2.69], [2.5792]
[2.41], [2.2439]
[3.02], [2.9743]
[3.2], [3.1899]
[2.77], [2.675]
[2.69], [2.5792]

MAE = [0.0897]

Fit # 4: Slope m = 0.834 Intercept b = 0.535 eV R2 = 0.811
[2.62], [2.5016]
[2.93], [2.8735]
[3.25], [3.2574]
[2.69], [2.5856]
[2.82], [2.7416]
[3.09], [3.0655]]

MAE = [0.0649]

Fit # 5: Slope m = 0.915 Intercept b = 0.333 eV R2 = 0.805
[2.62], [2.4997]
[3.33], [3.2755]
[3.04], [2.9586]
[3.09], [3.0133]
[2.77], [2.6636]
[2.54], [2.4123]

MAE = [0.0945]

Fit # 6: Slope m = 0.829 Intercept b = 0.555 eV R2 = 0.721

[2.35], [2.1654]
[3.25], [3.2509]
[3.2], [3.1906]
[2.42], [2.2498]
[2.74], [2.6358]
[2.79], [2.6961]

MAE = [0.0939]

Fit # 7: Slope m = 0.847 Intercept b = 0.518 eV R2 = 0.770
[2.37], [2.1854]
[2.74], [2.6221]
[2.36], [2.1736]
[2.53], [2.3743]
[2.62], [2.4805]
[2.41], [2.2327]

MAE = [0.1602]

Fit # 8: Slope m = 0.848 Intercept b = 0.502 eV R2 = 0.753
[3.09], [3.052]
[2.35], [2.1792]
[2.43], [2.2735]
[2.77], [2.6745]
[2.69], [2.5802]
[2.4], [2.2381]

MAE = [0.1221]
Fit # 9: Slope m = 0.882 Intercept b = 0.437 eV R2 = 0.772
[2.53], [2.3732]
[2.4], [2.2258]
[3.04], [2.9515]
[2.43], [2.2598]
[2.41], [2.2371]
[2.38], [2.2031]

MAE = [0.1566]

Fit # 10: Slope m = 0.843 Intercept b = 0.552 eV R2 = 0.792
[2.82], [2.6899]
[2.43], [2.2274]
[2.41], [2.2037]
[2.37], [2.1563]
[2.38], [2.1681]
[2.93], [2.8203]]

MAE = [0.1791]

Average values and standard deviation
Slope m = 0.864 +/- 0.032 Intercept b = 0.470 +/- 0.083 eV

S13 Cartesian coordinates and output calculations additional data

A data set collection of computational results is available in the ioChem-BD repository[1] and can be accessed https://www.iochem-bd.org/handle/10/390382

[1] Álvarez-Moreno, M.; de Graaf, C.; Lopez, N.; Maseras, F.; Poblet, J.M.; Bo, C. J. Chem. Inf. Model. 2015, 55, 95, 103.

S14 Statistical treatment of M-O and M-N bonds from structural data.

To check whether the bond description between the Y-O and Y-N and Eu-O Eu-N was providing with reasonable results, a search for these bond lengths was done on the Cambridge structural Database. Statistical data is shown in the following table, as well as the histogram distribution for each bond. Results are in excellent agreement with our computed bond lengths at the B3LYP-GD3BJ/def2TZVP method for geometry optimizations.

Bond	*Average value/Å*	*Standard deviation*	*Number of hits*	*Minimum value/Å*	*Maximum value/Å*
Y-O	2.348	0.107	16871	1.95	3.08
Y-N	2.422	0.128	8124	1.92	3.04
Eu-O	2.434	0.097	37347	1.91	3.28
Eu-N	2.590	0.086	9311	2.20	3.11

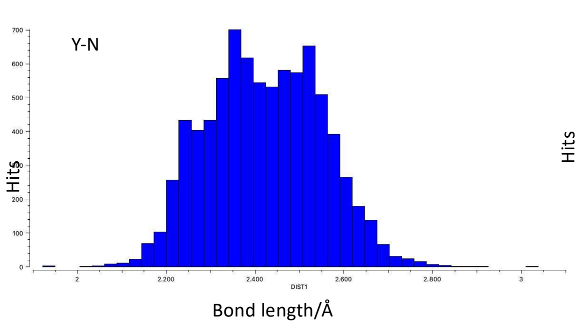

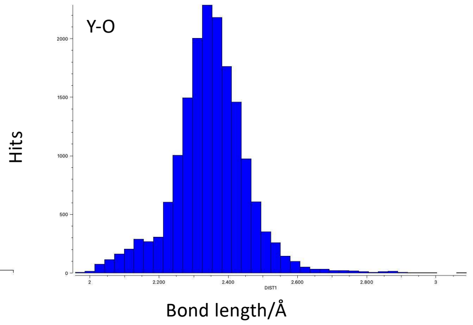

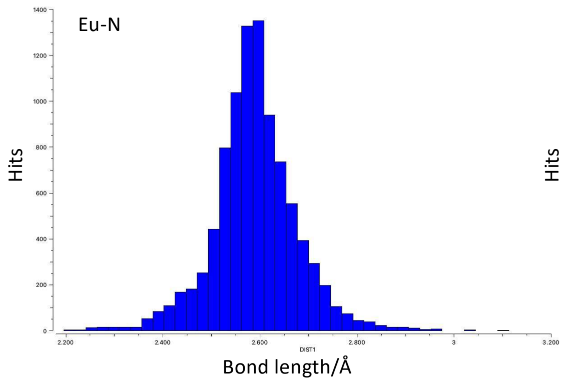

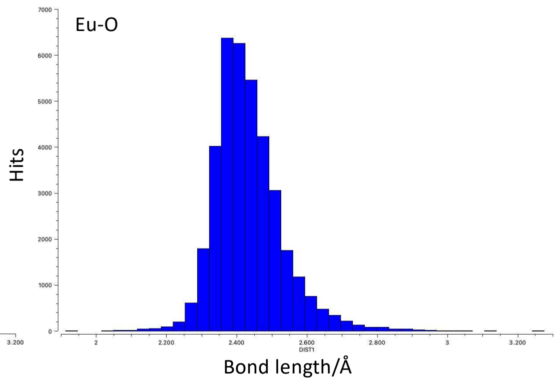

Supplement: Supplementary file 1 — Supplementary Material [file CPHC-27-e202500543-s001.zip › Supporting Information.pdf]
